# Death of Dr. A. H. McCord

**Published:** 1912-10

**Authors:** G. C. Priest


### Death of Dr. A. H. McCord.

This popular physician died August 29, 1912, aged 57. Dr. McCord was born in Fayette county, Texas, September 7, 1854. In the early part of 1865 both parents died, and in March, 1866, with two brothers and two sisters, he came to Houston county, and in December, 1869, he went to Rusk. His first work here was carrying mail from Rusk to Larissa for Mr. W. B. Boyd; worked at this seven or eight months and then went to work for Mr. H. Tribble at his sawmill four miles south of Rusk; worked with Mr. Tribble, saving his wages ($12 per month), paying his way to school to Prof. John Joss, who was principal of the old Rusk Masonic Institute. This was practically his first schooling. He taught school in the summer and attended school in the winter until he decided to study medicine. He commenced reading medicine under Dr. T. Y. T. Jameson and made a crop for him for the use of his books and his help. He attended his first course of lectures at the old Missouri Medical College in 1876, and returned home and taught school the next year; returned to college in the fall of 1878, and in the spring of 1879 he received his diploma. He returned to Rusk then and began the practice of medicine in the Atoy community, ten miles east of Rusk, and was married there to Miss Margarett E. Maness on June 20, 1881, and lived in that neighborhood until appointed Prison Physician by Governor Hogg in the fall of 1892, to fill the vacancy of Dr. W. T. Jameson, who resigned and moved to Palestine. He held this position during the administrations of Governors Hogg, Culberson, Sayers, Lanham, Campbell and Colquitt, with the exception of one year under Governor Sayers' administration. He was a stockholder and director in both of the Rusk banks; President of the Young Men's Business League and interested in several other public enterprises; was a member of the Presbyterian Church; was President of the County Medical Association for a number of years, and was also a member of the State Medical Association. He has not been actively interested in either of the Associations for the past fifteen years.

Very sincerely,

G. C. PRIEST.

